# Beverage consumption and obesity in Kuwaiti school children

**DOI:** 10.3389/fendo.2023.1174299

**Published:** 2024-03-11

**Authors:** Muhanad Alhareky, Jo Max Goodson, Mary Tavares, Mor-Li Hartman

**Affiliations:** ^1^ Department of Preventive Dental Sciences, Imam Abdulrahman Bin Faisal, College of Dentistry, Dammam, Saudi Arabia; ^2^ Department of Applied Oral Sciences, the Forsyth Research Institute, Cambridge, MA, United States; ^3^ The Forsyth Institute, Cambridge, MA, United States

**Keywords:** adolescent obesity, beverage, weight, BMI - body mass index, SSB products

## Abstract

Sweetened beverage consumption is particularly important in countries such as Kuwait, where the prevalence of obesity is high, and most children drink sweetened beverages daily. To assess the relationship between three most commonly consumed beverages, (soda, milk, and juice) and the incidence of obesity among Kuwaiti children at the critical age of 10-12 year, Longitudinal cohort data of 6,305 children on initial presentation in 2012 (age, 10 years) and follow-up in 2014 (age, 12 years) were obtained from the Kuwait Healthy Life Study. The servings for the three beverages (soda, juice, and milk) were calculated as servings per day groups (0, 1-2, and 3 servings/day or more). Multivariate logistic regression was performed to assess the relationship between developing obesity during 2012-2014 and soda, juice, and milk consumption. Model selection was based on clinically relevant covariates and potential confounders using stepwise model selection. Six percent children become obese between baseline and follow-up visits. High soda drinking showed significant association with developing obesity. High milk consumption (more than 3 servings a day) was also significantly associated with developing obesity. Potential confounders included in the final model were age, sex, governorates, and fitness level, of which none were significant confounders or effect modifiers for the association. Children with high soda consumption had significantly higher prevalence of obesity. High obesity prevalence was observed with high milk consumption at a lower significance level but not with high juice consumption.

## 1 Introduction

The effect of sugar-sweetened beverage (SSB) consumption on obesity development is an increasing healthcare concern (1). Although epidemiological data for adults reveal that sugar consumption is associated with type 2 diabetes (T2D) directly and indirectly (2), limited comparable data are available for children. Obesity and type 2 diabetes have a strong association. The risk and severity of type 2 diabetes are connected to body mass index (BMI). Obese people are seven times more likely to get diabetes than normal-weight people, whereas overweight people are three times more likely (3).

Nutrition evolution, fast socioeconomic developments, and westernization have all had a substantial impact on Kuwaiti adolescents’ lifestyle and dietary preferences during the last few decades, which has led in a significant shift in eating patterns, with fast food becoming an integral component of the Kuwaiti diet (4). Another study on Kuwaiti adolescents proposed the factors of weight gaining would be that Kuwaiti teenagers prefer high-energy snacks such as soft drinks and sugar-sweetened beverages, sweets, chocolate, potato chips, French fries, and fast food, which replace nutritious foods such as vegetables, whole grain products, and milk (5). Adolescents may gain weight if they consume unhealthy snacks instead of main meals on a regular basis.

Kuwait has one of the world’s highest percentage of adults with obesity (6, 7) and diabetes (8, 9). In 2005, a survey showed that 75.5% of Kuwaiti adults were overweight and 42.1% were obese (10), although these percentages were high, the more alarming observation was the rapid increase to 80.4% of overweight individuals and 47% of obese individuals for both sexes after 2 years (11). In 2009 a study reported, 10-14-year-old Kuwaiti children had an overall prevalence of 30.7% and 14.6% with respect to being overweight and obese (12).

In 2015, the World Health Organization (WHO) made several recommendations to reduce sugar intake, mainly because of its role in obesity and dental caries (13). Several studies have examined the relationship between SSB and obesity (14, 15). Soft drinks, in particular, have been assessed because they represent the primary source of added sugar in the diet, accounting for approximately 36% of the total added sugar consumed (16).

The dietary habits of Kuwaitis has major changed because of their lifestyle transformation, particularly after the Gulf War in 1990 (17). Honkala et al. showed that 13-year-old Kuwaiti children consume a higher proportion of soft drink consumption than children from 34 countries participating in their study (18). Approximately 75% of Kuwaiti children in that study consumed soft drinks every day (18). To our knowledge, no longitudinal studies have assessed the effect of beverage consumption of children on obesity development in the Middle East and North Africa (MENA) region (19).

With such an alarming percentage of obesity and high soft drink consumption, this longitudinal cohort study was conducted to assess the relationship between three most commonly consumed beverages, (soda, milk, and juice) and the incidence of obesity among Kuwaiti children over 2 years.

## 2 Methods

### 2.1 Subjects and study design

We obtained data of Kuwaiti children enrolled in Kuwait public schools. All participants were Kuwaiti nationals, and only 4^th^- and 5^th^ -grade Kuwaiti students were included in the study. Participants were selected to represent each of the six Kuwait governorates. All children provided a signed parent/guardian informed consent in Arabic, and assent for adolescents was obtained on the day of the school visit. The first school visit (baseline) occurred in 2012, in which 8,317 students participated. In 2014, 6,316 adolescents from the original sample enrolled in the follow-up visit. Longitudinal data were collected from this second group. Data from 11 subjects were excluded owing to incomplete information, and thus, the final sample size was 6,305 subjects. Both the Dasman Diabetes Institute Ethical Review Committee in Kuwait and the Forsyth Institutional Review Board reviewed and approved the study. The study used a longitudinal observational prospective analysis. We describe further details of the study elsewhere (20–22).

### 2.2 Beverage scoring

A questionnaire was prepared and administered in Arabic and English *via* iPads. Children were asked to select what they usually ate and drank with each meal and as a snack. The list of food items was based on responses from a pilot study conducted among 95 Kuwaiti schoolgirls before launching the survey (23). The dietary preference questions included 79 food and beverage items with accompanying pictures, and food selection options were modified to reflect the regularly consumed foods in Kuwait. Interviewers queried the children regarding the food items they usually ate for breakfast, lunch, dinner, and snacks. Following the questions on food preferences, questions on portions were presented, with pictures provided to assess the difference between portion sizes, e.g., one can, two cans, and three or more cans of soda with each meal. At the end of food selection, we asked the children if they preferred diet or regular soda and if they drink flavored or unflavored milk. The beverage for breakfast, lunch, dinner, and snacks was added to give a total number of servings. By this procedure, we obtained total servings per day for each of the three commonly used beverages (soda, juice, and milk). We excluded coffee and tea owing to a minimal number of subjects consuming these beverages. We computed the servings for each of the three beverages (soda, juice, and milk) as servings per day categories (0, 1-2, and 3 servings/day or more). Those who reported 0 serving a day were considered to have no consumption of the beverage. We analyzed consumption of drinks in three categories, i.e., non-consumers (0 serving/day), moderate consumers (1-2 servings/day), and high consumers (≥ 3 servings/day).

### 2.3 Obesity measurements

During the two visits, both weight and height were measured. Weight (kg) and height (cm) were used to compute the body mass index (BMI) of each participant. At each visit, we categorized the participants as either obese or nonobese utilizing the WHO definition of obesity with the use of BMI Z-score obesity cutoff (24). If the participant had a Z-score higher than 2 standard deviations, they were considered obese. Children were either obese or nonobese at baseline and follow-up. Based on these two visits, participants were placed in one of four groups, i.e., became obese, remained nonobese, remained obese, and became nonobese.

### 2.4 Data management and statistical analysis

At both visits, the four groups based on obesity status were used to identify children who developed obesity during the study period. The children who were nonobese at baseline and became obese at follow-up were identified as the “became obese group” (Group 1). Children in this group developed obesity during the two-year monitoring period. Children who were nonobese at baseline and remained nonobese at follow-up were identified as the “remained nonobese group” (Group 2). Children who were obese at baseline and remained obese at follow-up were identified as the “remained obese group” (Group 3). Children who were obese at baseline and became nonobese at follow-up were identified as the “became nonobese group” (Group 4). To analyze the effect of age, we divided the children into two groups, i.e., above and below the median age (9.9 years) at baseline visit. Fitness level was measured using the Queens College step test (25) as the increase in heart rate (beats/minute) following a standard exercise. The chi-square test was used to determine the significance level between the children who developed obesity and the other groups combined. Binary group differences in sex, age, and fitness level, along with consumption percentages of different levels of soda, juice, and milk consumed, were tabulated.

We analyzed the association between the three beverages consumed and developing obesity within the two-year study period using univariate logistic regression. The univariate logistic regression analysis was performed to assess the crude association between each beverage and the odds of developing obesity. To identify confounders, the following variables were tested separately using logistic regression based on clinical relevance and possibility of being confounders: age, sex, governorate, blood pressure, fitness level, salivary glucose level, and salivary high-density lipoprotein cholesterol (HDLC) level. None of these variables had an odds ratio (OR) of ≥10% difference compared to the crude association OR with any of the three beverages. We added variables to the stepwise selection model keeping only significant variables at a significance level of p = 0.05. Age, governorate, and fitness level were significant, but sex was not. All other tested variables were not statistically significant and therefore were not added to the completely adjusted multivariate model. We created interaction terms for all variables and found no significant effect modification. We examined the goodness of fit for all of the three beverages using the Hosmer-Lemshow test, and all three were found to have sufficient goodness of fit. The crude and completed adjusted models are found in **Table 1**. The children who developed obesity (Group 1) were the group of interest. We tested these children against all the other three groups combined. **Table 2** demonstrates the crude association and the fully adjusted model for Group 1 against each group separately, first against the “remained nonobese group” (Group 2) alone, followed by the “remained obese group” (Group 3) alone, and finally against the “become a non-obese group” (Group 4) alone. To test the trend between the categories for each beverage in both crude and adjusted models, we used the categorical beverage variable coded as a continuous variable. The p-value was ≤0.05 which showed a statistically significant trend between categories.

**Table 1:** Characteristics of Kuwaiti children by obesity change status from 2012 to 2014 using the chi-square test.

Variable	Those who became obese (Group 1, n = 378, 6%)^Δ^	Those who did not become obese (Group 2,3 and 4, n = 5927, 94%)^Δ^	P value
Sex			<0.001**
Female 3,958 (62.8%)	229 60.6%	3729 62.9%	
Male 2,347 (37.2%)	149 39.4%	2198 37.1%	
Age ^α^			<0.001**
Younger (≤9.9 years) 3,157 (50.1%)	217 57.4%	872 47.7%	
Older (>9.9 years) 3,148 (49.9)	161 42.6%	955 52.3%	
Fitness level ^β^			0.143
Low fitness level (≥23.5 bpm) 3,149 (49.94%)	175 46.3%	2,974 50.18%	
High fitness level (<23.5 bpm) 3,156 (50.06)	203 53.7%	2953 49.82%	
Soda consumption			0.001**
No soda 2,170 (34.4%)	130 34.39%	2040 34.4%	
Moderate soda consumption 3,649 (57.9%)	201 53.17%	3448 58.2%	
High soda consumption 486 (7.7%)	47 12.43%	439 7.4%	
Juice consumption			0.116
No juice 2,670 (42.3%)	158 41.8%	2512 42.4%	
Moderate juice consumption 2,640 (41.9%)	155 41.0%	2485 41.9%	
High juice consumption 995 (15.8%)	65 17.2%	930 15.7%	
Milk consumption			0.057
No milk 2,766 (43.9%)	157 41.5%	2609 44.0%	
Moderate milk consumption 3,322 (52.7%)	200 52.9%	3122 52.7%	
High milk consumption 217 (3.4%)	21 5.6%	196 3.3%	

p values listed are computed for the ≥3-serving group for soda, juice, and milk consumption. α = age divided into younger and older age groups around the median age at baseline (9.9 years). β = fitness level divided into low and high fitness level groups around the median fitness level at baseline (23.5 bpm). Δ = obesity as defined by WHO obesity Z-score. bpm = beats/minute.

* Significant (p ≤ 0.05) by chi-square test. ** significant (p ≤0.001).

**Table 2:** Logistic regression model for the relationship between children who developed obesity (Group 1) and those who did not (Groups 2, 3, and 4).

Variable	1. Crude			2. Adjusted		
	Group 1 vs. Groups 2, 3, and 4			Group 1 vs. Groups 2, 3, and 4		
	OR	95% CI	P-value	OR	95% CI	p value
Soda	Trend p value = 0.107			Trend p value = 0.111		
No soda	–	–	–	–	–	–
Moderate soda consumption	0.91	0.73-1.15	0.442	0.91	0.73-1.14	0.426
High soda consumption	1.68	1.18-2.38	0.004*	1.68	1.19-2.39	0.004*
Juice	Trend p value = 0.582			Trend p value = 0.552		
No juice	–	–	–	–	–	–
Moderate juice consumption	0.99	0.79-1.25	0.943	1.01	0.80-1.27	0.936
High juice consumption	1.11	0.82-1.50	0.489	1.11	0.82-1.50	0.494
Milk	Trend p value = 0.109			Trend p value = 0.110		
No milk	–	–	–	–	–	–
Moderate milk consumption	1.06	0.86-1.32	0.569	1.07	0.86 -1.32	0.565
High milk consumption	1.78	1.10-2.87	0.018*	1.77	1.10-2.87	0.019*

Adjusted values are for age, sex, governorate, and fitness level. OR = odds ratio. 95% CI = 95% confidence interval *Significant (p≤ 0.05).

A cross-sectional analysis of the baseline data was conducted using multivariate logistic regression analysis after adjusting for age, sex, governorate, and fitness level to compare it to longitudinal analysis.

## 3 Results

**Figure 1:**
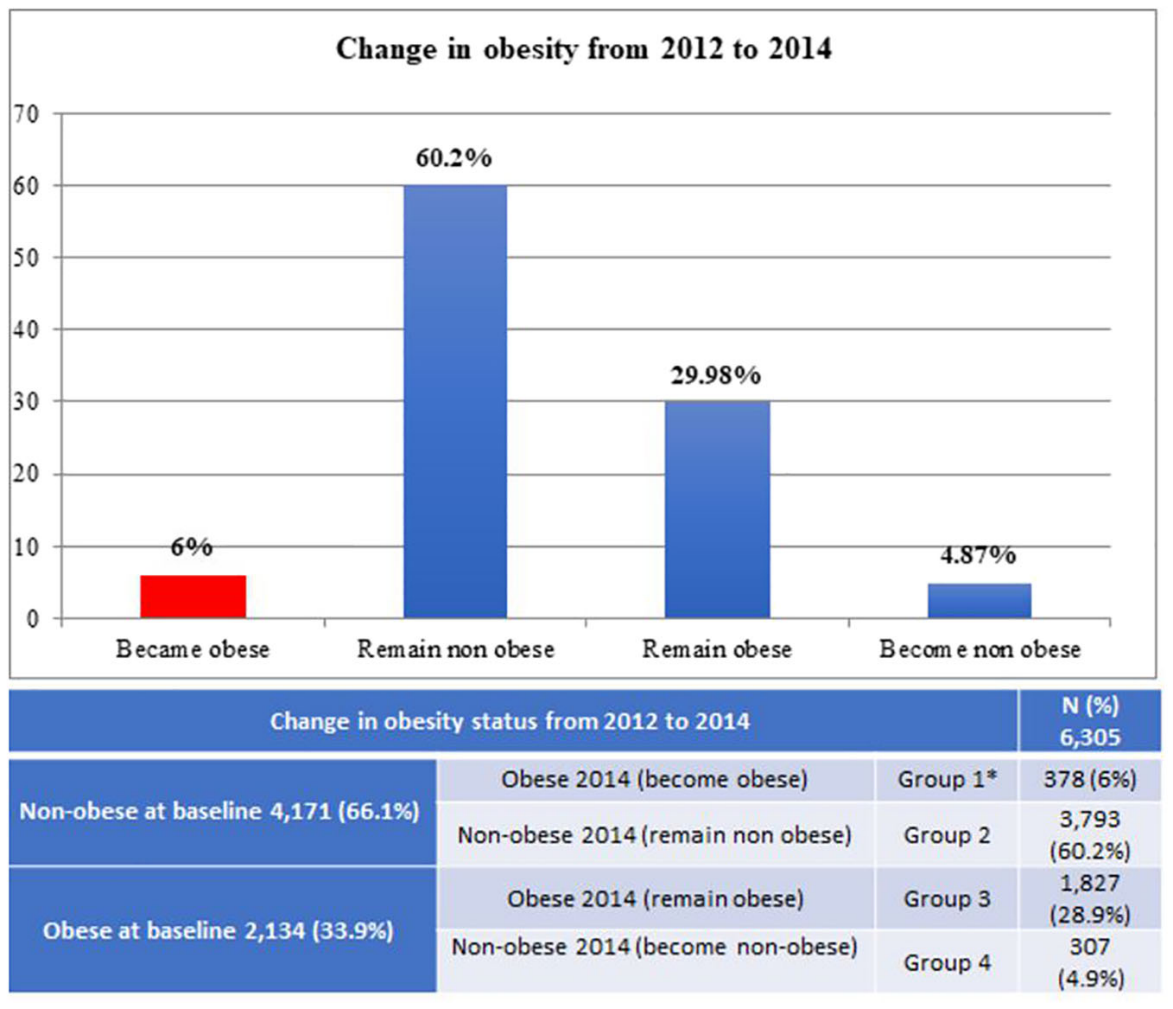
Change in obesity status from 2012 to 2014. Group 1 is the interest group. Children in Group 1 who developed obesity during the two-year study period. We compared the other groups with the interest group. *Interest group.

The study included 6,305 children (**Figure 1**). At baseline, 4,171 (66.1%) were non-obese and 2,134 (33.9%) were obese. The target population in Group 1 (n = 378, 6%) developed obesity between baseline and follow-up visits. Group 2 (n = 3,793, 60.2%) were non-obese at baseline and remained non-obese at follow-up visit. Group 3 (n = 1,827, 28.9%) were obese at baseline and remained obese at follow-up. Group 4 (n = 307, 4.9%) became non-obese at follow-up.

**Table 1** illustrates the characteristics of participants who became obese (Group 1) and those who did not (Groups 2, 3, and 4). Girls from all age groups, and children (boys and girls) less than 9.9 years old had a higher tendency to develop obesity (p < 0.001). Fitness level did not differ significantly between those who developed obesity and those who did not (p *= 0.143*). We found none of the comparisons of moderate consumption (1-2 servings) of juice and milk to be significantly associated with obesity. Children who developed obesity during the study period reported the highest percentage of individuals with high consumption of soda (12.43% compared to 7.4%, p = 0.001). By this analysis, high milk consumption was also not found statistically significant with the obese. (5.6% compared to 3.3%, p = 0.057). Children consuming juice did not differ significantly in the percentage of becoming obese (17.2% compared to 15.7%, p = 0.116).

The association of the different beverages and the odds of becoming obese (**Table 2**) demonstrated that participants who reported to have high soda consumption had an OR of 1.68 in becoming obese compared to those who reported without soda consumption (p = 0.004). Subjects who reported high consumption of milk had odds of 1.78 times than that of becoming obese compared to those who reported without milk consumption (p = 0.018). Adjustment for potential confounders did not alter this association between obesity and soda consumption. Milk had a similar effect, as those who reported with high consumption had an OR of 1.77 in becoming obese compared to those who reported with no milk consumption at all (p = 0.019). Consumption of juice (OR = 1.11) did not significantly affect the percentage of children who became obese (p = 0.494).

A comparison of the obese group (Group 1) with each of the other three groups is shown in **Table 3**. By this analysis, we found that children who reported high soda consumption showed significantly higher odds of being obese than those who reported with no soda consumption for every group comparison. We found the highest odds of becoming obese in those who became obese compared to the children who became non-obese at follow-up (OR = 2.42, p = 0.005). Milk was only significant when we compared children who became obese (Group 1) to children who were nonobese at both visits (OR= 1.87, p = 0.013). We did not find that consumption of milk was significantly associated with any of the other groups. Consumption of juice was also not significantly associated with any group. None of the tests in **Table 3** any significant trend.

**Table 3:** The association between consumption of three beverages and developing obesity over a two-year period.

Variable	Group 1 vs. Group 2			Group 1 vs. Group 3			Group 1 vs. Group 4		
	OR	95% CI	p value	OR	95% CI	p value	OR	95% CI	p value
Soda	Trend p value = 0.162			Trend p value = 0.174			Trend p value = 0.078		
No soda	–	–	–	–	–	–	–	–	–
Moderate soda consumption	0.91	0.72 -1.15	0.432	0.93	0.73-1.18	0.538	0.92	0.66- 1.28	0.615
High soda consumption	1.68	1.17-2.42	0.005*	1.58	1.08-2.32	0.019*	2.42	1.31-4.47	0.005*
Juice	Trend p-value = 0.646			Trend p value = 0.259			Trend p value = 0.891		
No juice	–	–	–	–	–	–	–	–	–
Moderate juice consumption	0.98	0.78-1.24	0.864	1.07	0.841.37	0.564	0.92	0.651.28	0.607
High juice consumption	1.06	0.78-1.43	0.720	1.21	0.87-1.67	0.257	1.1	0.69-1.74	0.699
Milk	Trend p value = 0.059			Trend p value = 0.318			Trend p value = 0.367		
No milk	–	–	–	–	–	–	–	–	–
Moderate milk consumption	1.08	0.87-1.35	0.489	1.01	0.80-1.27	0.932	1.05	0.76 -1.44	0.773
High milk consumption	1.87	1.14- 3.06	0.013*	1.63	0.96-2.77	0.070	1.68	0.75-3.74	0.205

This assesses children who developed obesity (Group 1) with each group, i.e., the groups comprising children who remained nonobese (Group 2), obese children who became nonobese (Group 3), and those who were obese and remained obese (Group 4) * Significantly associated at 0.05 level.

**Figure 2:**
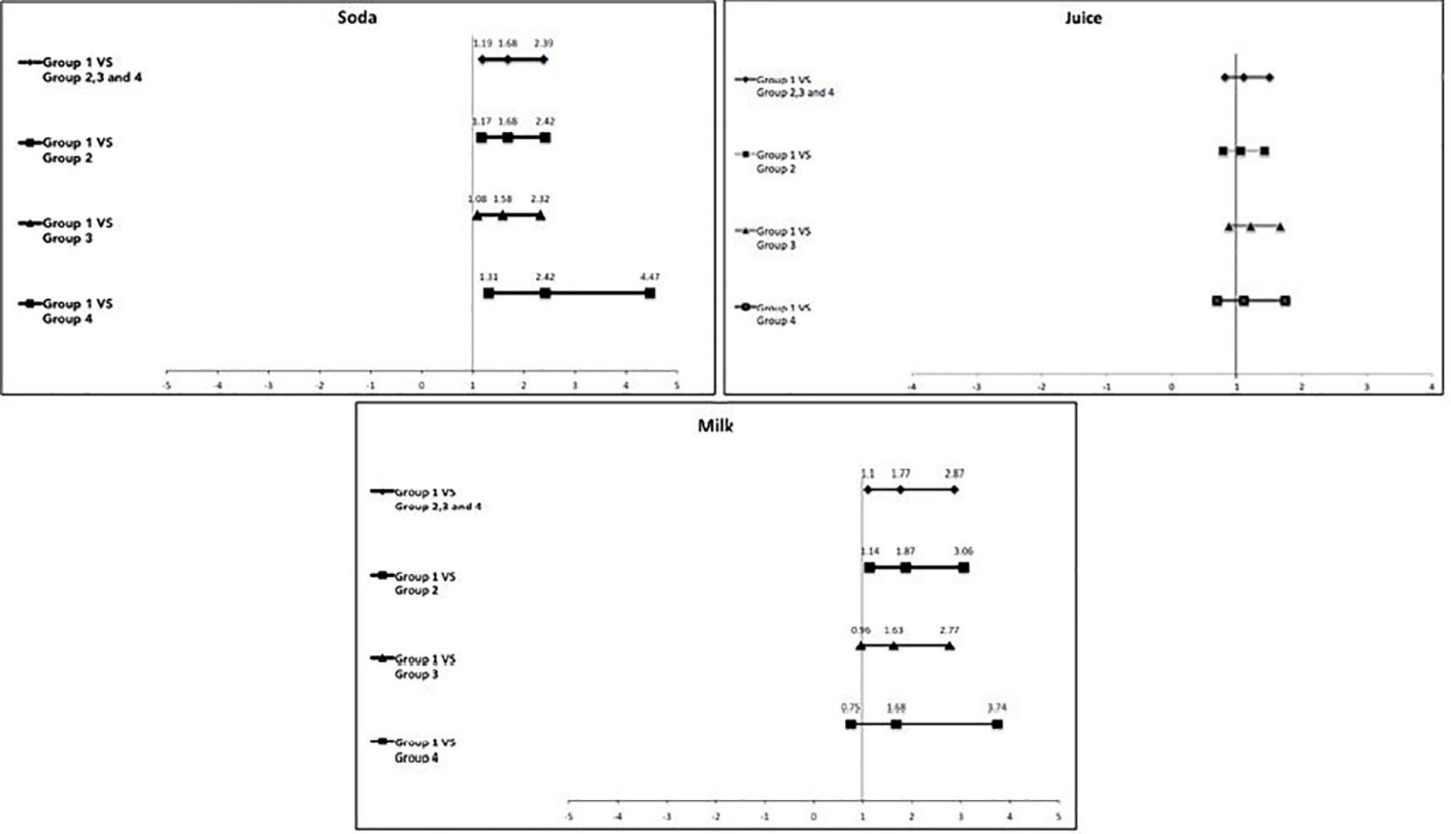
OR and 95% confidence intervals showing the association between high soda, juice, and milk consumption in children who developed obesity (interest group) and all the other three groups. If the bar passes through the solid vertical line, it means there is no significant association. Group 1: Became obese; Group 2: Remained nonobese; Group 3: Remain obese; Group 4: Became nonobese.

**Figure 2** summarizes the association between high consumption of any of the three beverages and the odds of being obese, comparing Group 1 to all and each of the other three groups.

## 4 Discussion

Our findings suggested that soda and to a lesser extent, milk but not juice consumption led to Kuwaiti children becoming obese (**Table 1**). Only high beverage consumption affected obesity. This association was only evident when we assessed longitudinal data but not cross-sectional data.

As per authors knowledge, this study is the first study to be done on grade 4^th^ and 5^th^ of school going children under big research that looks for salivary biomarkers. Some of the effects described in this analysis were likely associated with the climate in Kuwait. Most longitudinal observational studies are in North American (14, 26–34) and in European countries (35–38). Furthermore, we believe this is the first time that this association has been investigated in one of the MENA countries that have a high prevalence of both sugary drink consumption (18) and (11) obesity. The majority of studies investigating SSB and weight gain in children showed a positive association between the two (15). SSB includes a spectrum of beverages such as sugar-added soft drinks/sodas, energy drinks, flavored juice beverages, sports drinks, coffee and tea with added caloric sweeteners, and electrolyte replacement drinks (39). Some of the studies investigated these beverages combined as one group (14, 29, 31, 35), and some separated these beverages investigating each beverage separately accounting for a different source of sugar (25, 32, 33, 35). We found segregating the beverages to be beneficial as we found juice to be not associated with developing obesity, but soda and milk were. In other studies that segregated different SSB, soda was regularly associated with weight gain more than the other beverages investigated (32, 38). Striegel-Moore et al. (32) found that high soda consumption was the most reliable predictive factor of weight gain compared to other beverages including diet and regular soda, milk, coffee/tea, fruit juice, and fruit-flavored drinks. Viner et al. (38). also showed significant weight gain in children who reported high consumption of soda. Another study on young kids (5 years to 11 years) showed that the high frequency of SSB consumption had a roughly threefold increased chance of being overweight between the ages of 5/6 and 10/11 (40). Our study had the same finding that children with high soda consumption had higher odds of becoming obese. Soda is an excellent source of phosphate in diet, and high phosphate or phosphorus intake association with obesity has been reported in other epidemiological studies (41, 42), but the mechanism is still unclear (43). In our research group, Hartman et al. (44) found in a cross-sectional investigation of 77 children that salivary phosphate was significantly elevated in obese children compared to healthy weight children. Our data suggest that the combination of phosphorus with sugar in soda may synergize in the development of obesity. However, this hypothesis will require further investigation.

In contrast, high milk consumption resulted in children having significantly higher odds in becoming obese but only when the children were compared with those who were nonobese and remained nonobese (Group 2). Our findings concerning milk do not agree with findings from several longitudinal studies on its relationship with weight (45–47). Furthermore, some studies did not find any association between milk and changes in weight (48). On the other hand, our findings were consistent with the findings by Berkey et al. (49) as they found in a longitudinal study on 12,829 US children that drinking 3 or more servings of milk was associated with weight gain. In their findings, they also found that weight gain is attributed to the added calories from the milk because the association attenuated when they adjusted for the total energy intake. Another study on the different age group of children in Kuwait published recently, sweetened beverage consumption was linked to being overweight and having a higher BMI-for-age z score, respectively (19). In our study, we were unable to adjust for the total energy intake to identify if the association between milk and weight gain would remain the same or would disappear. Similar to the suggested contribution of phosphorus in the soda, we also, may suggest as a hypothesis that the fat content in milk in combination with added sugar would have contributed to the association between weight gain and obesity. Strong recommendations to limit sugar intake for adults and children were published by WHO (11) in 2015 due to its widely documented association with obesity and dental caries. We find it valuable to investigate some other ingredients added in some beverages that make them more associated with obesity. Phosphorus in soda beverages and fat in flavored or unflavored milk drinks are two good examples of some of these additives.

Considering that juice consumption is described to be associated with obesity by many (50), it was a surprise that compared to soda and milk, fruit juice was not found to have a significant effect on obesity. However, our findings on fruit juice are in agreement with other studies investigating fruit juice and obesity, Gustavo in 2017 concluded that moderate consumption of fruit juices has a lower risk of metabolic syndrome (51).

A vast majority of studies concerning dietary intake are usually self-reported; therefore, they are subject to reporter accuracy, bias, and recall especially in children and children (52). Our study is no exception, although we believe that using pictures for selection of both the beverages and portion size may help in improving the accuracy. As bottles, cans, and cups come in different sizes, the servings computed in the survey represent a proxy for the expected consumption pattern.

The questions about beverage consumption did not discriminate between regular and diet soda, but in a follow-up question at the end of the survey, children were asked if they chose regular or diet soda, and only 283 children (4.5%) reported drinking diet soda. Hence, we decided to keep these 283 children in the sample due to their small number. Conversely, another research on preschool children to investigate the connection of beverage intake with BMI revealed that higher beverage consumption (Milk, Soda, fruit drinks and fruit juices) correlated with an increase in the children’s overall calorie intake rather than their BMI (53).

Questions regarding milk did not inquire about the type of milk consumed (whole, low fat, skim, flavored, or unflavored). However, at the end of the survey, children were asked if they drink flavored milk and 3,152 (49.9%) children responded positively. Therefore, it is important to include flavored milk (with added sugar) in the milk category.

## 5 Conclusions

High consumption (3 or more servings) of soda and to a lesser degree, milk but not fruit juice was significantly associated with obesity development in Kuwaiti children, thus clearly indicating that there is more to obesity than simple sugar consumption. Neither high nor moderate juice consumption was significantly associated with obesity. Only high soda or milk consumption (≥3 servings/day) was associated with increased prevalence of obesity. Consumption of moderate amounts of any of these beverages (1-2 servings/day) was not associated with significantly increased prevalence of obesity.

**Figure 3:**
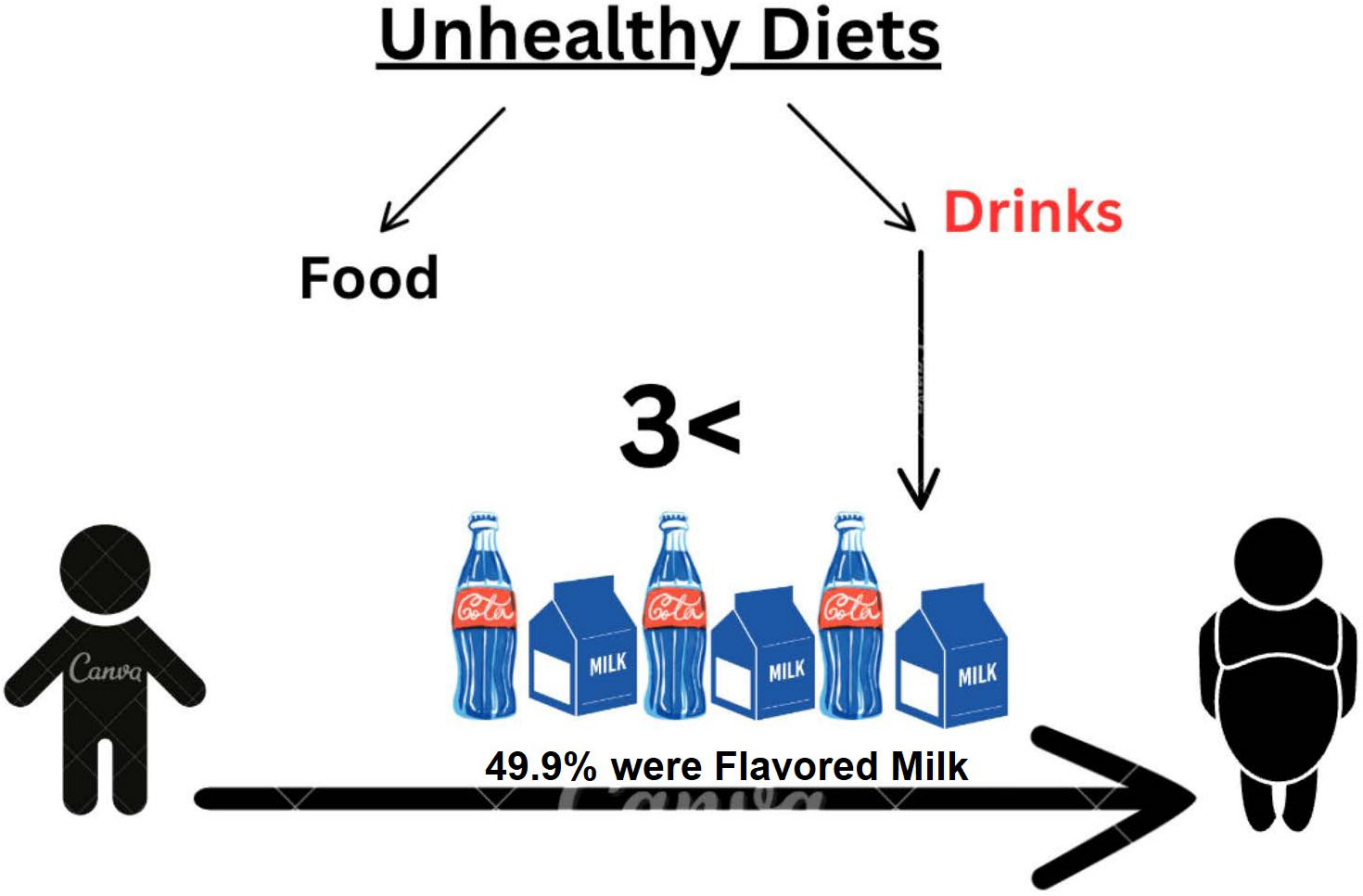
Representation of higher consumption.

In our results, we were able to find significant association between high soda and high milk (49.9% were flavored milk) consumption with developing obesity in Kuwaiti children. **Figure 3** is presenting that the higher consumption of sugar sweetened milk (49.9% were flavored milk) and soda may lead to the obesity.

## Data availability statement

The raw data supporting the conclusions of this article will be made available by the authors, without undue reservation.

## Ethics statement

The studies involving humans were approved by Both the Dasman Diabetes Institute Ethical Review Committee in Kuwait and the Forsyth Institutional Review Board reviewed and approved the study. The studies were conducted in accordance with the local legislation and institutional requirements. Written informed consent for participation in this study was provided by the participants’ legal guardians/next of kin.

## Author contributions

MA, MH, and JG designed and conducted the study. MA and MT analyzed the data. MA wrote the paper with the assistance of all authors. All authors contributed to the article and approved the submitted version.
